# Molecular Identification of *Spirometra erinaceieuropaei* Tapeworm in Cases of Human Sparganosis, Hong Kong

**DOI:** 10.3201/eid2304.160791

**Published:** 2017-04

**Authors:** Tommy H.C. Tang, Samson S.Y. Wong, Christopher K.C. Lai, Rosana W.S. Poon, Helen S.Y. Chan, Tak Chiu Wu, Yuk-Fai Cheung, Tak-Lap Poon, Yi-Po Tsang, Wai-Lun Tang, Alan K.L. Wu

**Affiliations:** Queen Elizabeth Hospital, Hong Kong, China (T.H.C. Tang, C.K.C. Lai, H.S.Y. Chan, T.C. Wu, Y.-F. Cheung, T.-L. Poon);; The University of Hong Kong, Hong Kong (S.S.Y. Wong, R.W.S. Poon);; Pamela Youde Nethersole Eastern Hospital, Hong Kong (Y.-P. Tsang, W.-L. Tang, A.K.L. Wu)

**Keywords:** sparganosis, *Spirometra*, Hong Kong, China, parasites, zoonoses, food safety, frog meat, snake meat, contaminated water, *Spirometra erinaceieuropaei*

## Abstract

Human sparganosis is a foodborne zoonosis endemic in Asia. We report a series of 9 histologically confirmed human sparganosis cases in Hong Kong, China. All parasites were retrospectively identified as *Spirometra erinaceieuropaei*. Skin and soft tissue swelling was the most common symptom, followed by central nervous system lesions.

Sparganosis is a parasitic zoonosis endemic in Asia, Europe, and North America. Diphyllobothroid tapeworm under the genus *Spirometra* is the causative agent. Humans can be infected through the consumption of contaminated water or meat from intermediate hosts or through topical application of raw, contaminated poultices to eyes and open wounds. After entry into humans, the plerocercoid larvae (spargana) migrate to different anatomic locations, where they cause space-occupying lesions as they develop into adults. The sites spargana migrate to include skin and soft tissues, muscles, visceral organs, and the central nervous system. Clinical symptoms range from asymptomatic/mild (e.g., subcutaneous swelling) to severe (e.g., seizure and hemiparesis) depending on the site and size of lesions (*1*).

Sparganosis is an emerging zoonotic disease and public health challenge in China, potentially because of the practice of consuming wild frog meat, which is a delicacy in the southern Guangdong province. According to a 2009 survey, >25% of the local wild frogs were infected with spargana (*2*). Most cases of human sparganosis have been found in Asia, with the highest cumulative number in China (Technical Appendix Table) (*3*). In Hong Kong, the earliest known cases of sparganosis were 2 subcutaneous infections reported in 1962 (*4*), and cases afterward have been sporadic. With advances in molecular sequencing, the identification of sparganum larvae isolated from humans was made possible (*5*,*6*). In this study, we performed molecular sequencing on archived histologic specimens to delineate the parasites down to species level.

## The Study

Cases of human sparganosis were identified by searching the clinical, parasitologic, and histopathologic records in the Queen Elizabeth Hospital and the Pamela Youde Nethersole Eastern Hospital in Hong Kong. Archived histopathology specimens showing parasites compatible with plerocercoids were retrieved for further molecular testing. We made 10–15 (depending on the amount of tissue available) 4-µm sections from each paraffin block; the sections were deparaffinized and suspended in sterile, normal saline. Genomic DNA was extracted from formalin-fixed paraffin-embedded tissue by using a DNA minikit (QIAGEN, Hilden, Germany) according to the manufacturer’s instructions. The DNA was eluted in 60 µL of elution buffer and used as template for PCR.

Primer sequences used in this study were cox1-F 5′-CGGCTTTTTTTGATCCTTTGGGTGG-3′, cox1-R 5′-GTATCATATGAACAACCTAATTTAC-3′, 28S-F 5′-CACCGAAGC CTGCGGTA-3′, and 28S-R 5′-GAAGGTCGACCTGGTGAA-3′, which targeted specifically to the *cox1* and 28S rRNA genes of *S. erinaceieuropaei* respectively (*7*). The later primers were designed in-house by multiple alignments of different parasite species. The PCR mixture (25 µL) contained DNA, PCR buffer (10 mmol/L Tris-HCl [pH 8.3], 50 mM KCl, 3 mmol/L MgCl_2_, and 0.01% gelatin), and 200 mmol/L each deoxynucleoside triphosphate (dNTP) and 1.0 U *Taq* polymerase (Applied Biosystems, Foster City, CA, USA). The mixtures were amplified in 60 cycles of 94°C for 1 min, 55°C for 1 min, and 72°C for 1 min with a final extension at 72°C for 10 min in an automated thermal cycler (Applied Biosystems). Standard precautions were taken to avoid PCR contamination, and no false-positive results were observed in negative controls. PCR products were gel purified by using the QIAquick gel extraction kit (QIAGEN). Both strands of the PCR products were sequenced twice with an ABI Prism 3700 DNA analyzer (Applied Biosystems). Sequences of the PCR products were compared with known sequences by BLAST analysis (https://blast.ncbi.nlm.nih.gov).

We constructed a phylogenetic tree using the neighbor-joining method with Kimura’s 2-parameter correction with ClustalX 1.83 (http://www.clustal.org). We included in the analysis the 252 bps and 211 bps of the amplicon from the *cox1* gene (GenBank accession nos. KU760072–81) and the 28S rRNA gene (accession nos. KX831668–77) of *S. erinaceieuropaei*, respectively, detected in positive samples. *Strongyloides stercoralis* was used as the outgroup in these analyses.

Seven patients with human sparganosis were identified in Queen Elizabeth Hospital, and 2 patients were identified in the Pamela Youde Nethersole Eastern Hospital. All diagnoses were made from 1999 to 2015 (Table). Eight patients were Chinese; 1 was Filipino, and 4 were male. Patient age at diagnosis was 29–73 (median 49) years. Three patients displayed neurologic symptoms, such as numbness, weakness, or memory impairment, and the other 6 displayed skin and soft tissue involvement. All had progressively enlarging or migratory skin nodules (Table). Additional information on clinical history, histopathology, and magnetic resonance brain imaging of representative cases was collected (Technical Appendix). 

**Table:** Characteristics of cases of human sparganosis, Hong Kong, 1999–2015*

Pt no.	Year	Age, y/sex	Ethnicity	Probable place/mode of infection	Location of lesion	Size of worm or lesion, cm	Clinical features	PEC, × 10^9^/L (% total WBC count)
1	1999	67/F	Chinese	Unk/Unk	Right breast	0.15 × 0.1 × 0.7, 0.15 × 0.1 × 0.7, 0.1 × 0.5 × 0.5 (lesions excised)	Right breast mass	NR
2	2000	46/M	Chinese	Unk/Unk	NR	0.15 (worm length)	NR	NR
3	2002	29/F	Chinese	Unk/Unk	Epigastrium of abdominal wall	4 × 2.5 × 2 (lesion excised)	NR	NR
4	2003	63/F	Chinese	Unk/Unk	Left thigh	0.6 (maximum dimension of lesion excised)	Progressive enlarging mass for 2 years	NR
5	2004	44/M	Chinese	Unk/Unk	Right thigh	1.5 × 1.5 (lesion); 0.27 × 0.2 × 0.5 (worm)	Right thigh nodule for 6 months	NR
	2014	55/M		Unk/Unk	Right thigh and suspected left frontal lobe	1.6 × 1.3 × 1.4 (lesion)	Recurrent right thigh nodule; suspicious 2 × 5 × 5 mm T2W/FLAIR hyperintensity with contrast enhancement in left frontal white matter	0.22 (3.7)
6	2005	43/F	Chinese	Unk/Unk	Left breast	0.21 (lesion excised)	Progressive enlarging left breast mass	0.1 (0.7)
7	2011	58/M	Chinese	China/ingestion of frogs and snakes	Left chest wall	3 × 2.5 × 1 (lesion)	Left chest wall mass for 3 years	0.21 (2.5)
8	2013	49/F	Filipino	Unk/Unk	Left parietal lobe	0.17 × 0.12 × 0.23 (lesion)	Right-sided numbness and weakness for 2 days	0.1 (1.1)
9	2015	73/M	Chinese	China/ingestion of frogs	Left thigh	0.5 × 0.5 × 0.1 (lesion excised)	Progressive enlarging left inner thigh mass for 1 year	0.21 (4.2)

*All worms were identified as *Spirometra erinaceieuropaei*. NR, not recorded; PEC, peripheral eosinophil count; Pt, patient; T2W/FLAIR, T2-weighted/fluid attenuation inversion recovery; Unk, unknown; WBC, white blood cell.

Nine patients had archived histopathologic specimens available for molecular testing. Parasite identification was achieved in all 9 specimens, and they showed 99%–100% and 100% identity with the *cox1* and 28S rRNA gene sequences of *S. erinaceieuropaei*, respectively (Figure, panels A and B).

**Figure:**
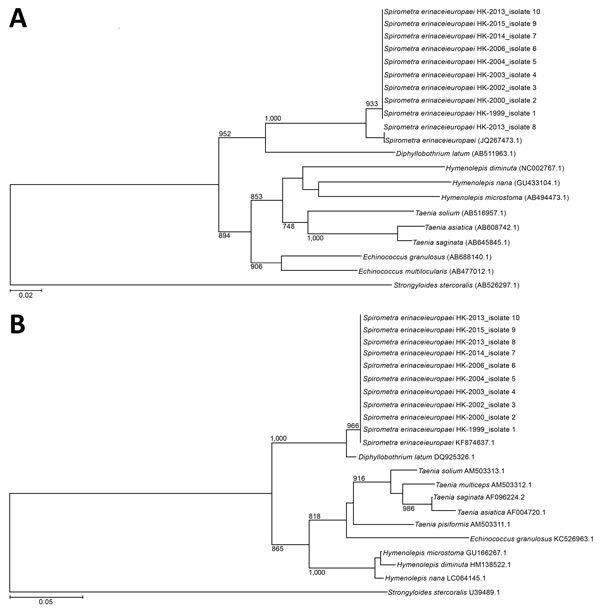
Phylogenetic analysis of *cox1* and 28S rRNA genes of archived formalin-fixed paraffin-embedded tissues obtained from human sparganosis cases, Hong Kong, 1999–2015. A) A 252-bp sequence from the *cox1* gene (GenBank accession nos. KU760072–81) was included for each isolate. B) A 211-bp sequence from the 28S rRNA gene (accession nos. KX831668–77) was included for each isolate. Trees were constructed by using the neighbor-joining method and rooted with the corresponding sequence in *Strongyloides stercoralis* (accession nos. AB526297.1 and U39489.1 for *cox1* and 28S rRNA genes, respectively). The bootstrap values are shown for nodes that appeared in >70% of the 1,000 replicates. The species used for comparison and their GenBank accession numbers are given in the tree. Scale bars indicate estimated number of substitutions per 50 bases.

## Conclusions

This study demonstrates that human sparganosis appeared sporadically in Hong Kong. The most common signs of disease were skin and soft tissue nodules followed by intracranial lesions. By molecular sequencing, the tested parasites were *S. erinaceieuropaei.* We were unable to pinpoint the source of infection in most patients; the incubation period can last as long as several months, and early stages of the disease are usually asymptomatic (*8*). Patients might have difficulty recalling specific high-risk exposures. In most industrialized countries, the practice of applying raw frog or snake poultices to open wounds is regarded as unhygienic and becoming obsolete, yet consumption of undercooked frog meat or, less commonly, ingestion of raw snake bile for medicinal purposes is still practiced in Hong Kong. Another possible route of transmission could have been drinking water contaminated with *Spirometra* procercoids.

Subcutaneous sparganosis is the most commonly recognized form of the disease. Because sparganosis is rare, it is seldom considered during an initial patient assessment, although a migratory nodule might raise the suspicion for a helminthic etiology. Diagnosis of sparganosis needs to be confirmed, normally by studying the excised lesions. Even though serologic tests for sparganosis have been described, these assays are not generally available and their performance requires more evaluation (*9*–*13*). In contrast, the presence of tunnel sign, conglomerated rings, bead-shaped enhancements, or images of parasites of various life stages by computerized tomography or magnetic resonance imaging are suggestive of sparganosis (*14*). Histopathologic diagnosis of parasitic infections remains a challenge to pathologists in countries where sparganosis is not endemic. Recognizing the different phyla and classes of parasites (i.e., nematodes, cestodes, and trematodes) histologically is usually simple. However, specific identification of the genus and species requires substantial expertise in parasite pathology and morphology. Identification of rare parasites is sometimes impossible because of the lack of detailed morphologic descriptions in the literature. Under such circumstances, molecular studies provide useful information for species identification (*15*). Nevertheless, it is not infallible, especially for rare parasites, because precise species identification depends on gene sequence availability and data accuracy.

Although the parasitic drug praziquantel has wide coverage against several cestodes and trematodes, its efficacy in the treatment of sparganosis remains uncertain. Surgical intervention for complete worm removal should be used whenever feasible.

This study had limitations. We only included information on patients from 2 of the 7 geographic clusters of public hospitals in Hong Kong, and those with asymptomatic subcutaneous lesions most likely did not seek medical attention. The reported number is certainly an underestimate. 

Given that human sparganosis is an emerging zoonotic parasitic infection, clinicians may consider it in the differential diagnosis for mass lesions with undetermined etiology. Education of the general public about food safety, including avoiding the consumption of untreated water and undercooked frog and snake meat, is needed.

Technical Appendix Description of sparganosis patients 4–8, with hematoxylin-eosin stainings of the lesion in patients 7 and serial magnetic resonance imaging scans of cranial lesions in patients 8, and a table summary of 12 previously published human sparganosis cases in Hong Kong.
