# Supplementary material for: Molecular Identification of Spirometra erinaceieuropaei Tapeworm in Cases of Human Sparganosis, Hong Kong
Source: Emerg Infect Dis. 2017 Apr;23(4):665–8. doi: 10.3201/eid2304.160791 (PMC5367436; doi:10.3201/eid2304.160791)
Supplement: Technical Appendix — Description of sparganosis patients 4–8, with hematoxylin-eosin stainings of the lesion in patients 7 and serial magnetic resonance imaging scans of cranial lesions in patients 8, and a table summary of 12 previously published human sparganosis cases in Hong Kong. [file 16-0791-Techapp-s1.pdf]

Article DOI: http://dx.doi.org/10.3201/eid2304.160791

Molecular Identification of *Spirometra erinaceieuropaei* in Cases of Human Sparganosis, Hong Kong

Technical Appendix

History of Representative Patients

Patient 4

A 63-year-old Chinese woman had a slow-growing mass in her left thigh for 2 years. Physical examination revealed an induration of a 2-cm diameter at the affected area. Microscopic examination of the sections from the excised lesion showed a mixed septal and lobular panniculitis surrounded by palisade of histiocytes and eosinophils. Histologic cross-sections showed viable parasite with folded teguments having brush border, smooth muscles, and calcareous corpuscles inside. No alimentary tract or hooklets could be found. Patient was cured by surgery without recurrence of disease.

Patient 5

A 44-year-old Chinese man had a right thigh nodule for 6 months in 2004. During excisional biopsy, a 1.5 cm × 1.5 cm lipomatous, subcutaneous nodule was removed. Fragments of a worm-like organism of ≈0.5–1 mm in breadth were noted on microscopic examination. The margin of the biopsy was involved. The worm possessed a tegument, beneath which were parenchyma containing additional tegument cells. The parenchyma also contained layers of smooth muscle tissue and calcareous corpuscles. No scolices or hooklets were identified. The subcutis contained a palisade of granulomatous inflammation. On the basis of these morphologic features, a provisional diagnosis of sparganosis was made. Because the margin was involved, a follow-up magnetic resonance imaging (MRI) scan was performed 1 month after surgery. It showed focal skin thickening with mild gadolinium

enhancement subjacent to the surgical scar. Otherwise the images were clear of signs of disease. Another follow-up scan was performed in 2008; this image indicated that the previous gadolinium enhancement that was adjacent to the site of the surgical scar was largely resolved.

In 2014, the patient noted a solitary mass reappearing over the previous surgical site. A small lesion was palpable. MRI showed an ill-defined, contrast-enhancing area of 1.6 cm located over the subcutaneous area of the anteromedial aspect of the right thigh. Microscopic examination of the tissue obtained from a wide excision of the lesion showed multinucleated giant cells surrounding a parasite. An MRI brain scan was performed and showed a 2.0 × 5.0 × 5.0 mm T2-weighted/fluid attenuation inversion recovery hyperintensity with contrast enhancement in the left high frontal white matter. There was no perifocal edema. The lesion remained static on subsequent MRI scan.

Patient 6

A 43-year-old Chinese woman had a nonpainful mass in her left breast. Physical examination showed a 1.5-cm nodule at the 2 o'clock position, 9 cm from the nipple. Ultrasound revealed a hyperechoic area ≈2.1 cm in diameter with ill-defined margins in the left breast. The lesion was subsequently excised; histologic staining of the lesion revealed necrotizing granulomatous inflammation with epithelioid cells, chronic inflammatory cells, and occasional eosinophils. There were no parasites seen in the excised specimen. The patient remained well after surgery without recurrence. Based on patient clinical history and the presence of eosinophils on histology, the clinician sent the tissue for a nucleic acid amplification test.

Patient 7

A 58-year-old Chinese man had a mass in his left chest for 3 years. There was no history of trauma. The mass caused mild pain and pruritus without systemic symptoms. The patient noted some migration of the mass over the years. Further questioning revealed that the patient had a history of frequent travel to mainland China, and he consumed snake and frog meat during these travels. Initial physical examination revealed a firm 1-cm chest wall mass. Results from fine-needle aspiration cytology showed suspected parasitic elements. During excisional biopsy, a 3.0 × 2.5 × 1.0–cm lesion was removed. Postoperative ultrasound showed no residual lesions.

Histopathology showed patchy, suppurative granulomatous inflammation containing a parasite, which contained muscle bands and spherical, dark-staining calcareous corpuscles with whorled appearance scattered in its parenchyma (online Technical Appendix Figure 1, panels A–C). The overall morphology was compatible with sparganosis. Ophthalmologic assessment and computer tomography scans of the brain showed no abnormalities. The patient was well 2 years after surgery with no evidence of disease recurrence.

Patient 8

A 48-year-old Filipino woman reported right-sided weakness and numbness for 2 days. On examination her upper and lower limb power was 4/5 (Medical Research Council scale for muscle power: https://www.mrc.ac.uk/research/facilities-and-resources-for-researchers/mrc-scales/mrc-muscle-scale/). An initial contrast computer tomographic brain scan showed a suspicious ring-like, heterogeneous enhancement at the high-left fronto-parietal region of the brain peripheral to the vertex with an associated perifocal white matter edema at the left corona radiate. The features were highly suspicious of a focal aggressive lesion either at the cortical region or the leptomeningeal region. A contrast MRI brain scan showed 3 enhancing intra-axial lesions in the left cerebral hemisphere (online Technical Appendix Figure 2, panels A–F). The largest one was seen in the high-left parietal, measuring 1.7 × 1.2 × 2.3 cm, and 2 others were noted in high-left parietal and left inferior frontal region with surrounding edema. The radiologic findings were suspected cerebral metastases. Open brain biopsy was performed and showed a thin layer of subdural tissue connected to the left parietal parenchymal lesion, a rubbery parietal lesion having a small amount of whitish discharge inside, and a large cortical vein adhering to the posterior edge of the parietal lesion. Intraoperative frozen-section of the sampled brain tissue showed necrotic and fibrous tissue with infiltrates of lymphocytes, plasma cells, and many eosinophils. Gram, Grocott, periodic acid-Schiff with diastase (PASD), and Ziehl-Neelsen (ZN) stains were negative for microorganisms. Histology of the brain tissue showed necrotic material surrounded by a granulomatous reaction, with chronic inflammatory infiltrates and some eosinophils. There was no evidence of malignancy. A necrotic helminth was identified within the necrotic area. Due to the extensive necrosis, initial identification of the

helminth was deemed impossible. Oral albendazole 400 mg twice daily and dexamethasone 2 mg 4 times daily were given for 2 weeks.

The patient's right-sided numbness persisted. A follow-up MRI brain scan 62 days after the brain biopsy showed that the heterogeneous lesions in the high-left parietal regions were still present and had migrated (online Technical Appendix Figure 2, panels G and H). The scan showed a lesion with a serpentine, elongated configuration and a T2-hypointense signal with hyperintense center together with heterogeneous rim/nodular contrast enhancement. The more anterior lesion appeared larger in size and measured ≈0.8 × 0.8 cm in cross-sections and 1.8 cm in length. It showed deeper involvement into the subcortical white matter. Perifocal T2-hyperintense signal was similar in extent. The posterior lesion was similar in size, measuring 0.1 × 0.6 × 0.2 cm. Perifocal T2-hyperintense signal was more extensive. The left frontal lesion ≈0.7 mm in size showed contrast enhancement with perifocal edema of similar extent. Oral praziquantel 1,500 mg 3 times daily and dexamethasone 2 mg twice daily were given for 1 week after diagnosis of sparganosis was confirmed by molecular sequencing.

The patient had a generalized tonic-clonic seizure 90 days after brain biopsy. The follow-up MRI brain scan 103 days after brain biopsy showed increased caudal extent with deeper involvement into the subcortical white matter for the left frontal lesion. The patient refused to repeat brain surgery for complete excision and received 2 weeks of oral praziquantel 1,500 mg and cimetidine 400 mg 3 times daily. She returned to the Philippines afterwards with residual right-sided limb power of 4+/5.

References

1. Ng TH, Wong WT, Fung CF, Leung CY. Clinical sparganosis in Hong Kong. J R Soc Health. 1989;109:138–40. PubMed http://dx.doi.org/10.1177/146642408910900409

2. Huang CT, Kirk R. Human sparganosis in Hong Kong. J Trop Med Hyg. 1962;65:133–8. PubMed

3. Wong W, Huang C. A case of ocular sparganosis in Hong Kong. Far East Med J. 1970;1:107–9.

4. Chan S-T, Tse CH, Chan YS, Fong D. Sparganosis of the brain. Report of two cases. J Neurosurg. 1987;67:931–4. PubMed http://dx.doi.org/10.3171/jns.1987.67.6.0931

5. Fung CF, Ng TH, Wong WT. Sparganosis of the spinal cord. Case report. J Neurosurg. 1989;71:290–2. PubMed http://dx.doi.org/10.3171/jns.1989.71.2.0290

6. Chuen-Fung TL, Alagaratnam TT. Sparganosis of the breast. Trop Geogr Med. 1991;43:300–2. PubMed

7. Aung TH, Lee MK, Kwok JCK, Leung SCL. How safe is computed tomography-guided stereotaxy in neurosurgery and how should we select patients? Hong Kong Med J. 1995;1:329–34.

8. Kay R; Hong Kong Neurological Society. Casebook of neurology. Philadelphia: Lippincott, Williams & Wilkins; 2002.

9. Chan ABW, Wan SK, Leung S-L, Law BKB, Lai DPY, Ip M, et al. Sparganosis of the breast. Histopathology. 2004;44:510–1. PubMed http://dx.doi.org/10.1111/j.1365-2559.2004.01831.x

Table. Characteristics of previously reported human sparganosis patients*

Patient	Year	Age, y, sex	Ethnicity	Probable place/mode of infection	Location of lesion	Size of worm, L × W, lesion, L, cm	Clinical features	% peripheral eosinophils over total leukocytes	Reference
1	1962	30, F	Chinese	NR/undercooked frog meat	Chest wall	3.0 × 0.1–0.2	Subcutaneous mass	Normal	(*1*,*2*)
2	1962	2.5, M	NR	Hong Kong/undercooked frog meat	Abdominal wall	NR	Subcutaneous mass	9%	(*1*,*2*)
3	1970	32, M	Chinese	China/application of raw frog meat	Right eye	8.0 × 1.5–2.0	Migratory subconjunctival swelling	4%	(*1*,*3*)
4	1987	12, F	Chinese	China/contaminated water	Brain (right frontal)	NR	Convulsion	Normal	(*1*,*4*)
5	1987	56, M	NR	China/contaminated water	Brain (right parietal)	NR	Progress limb weakness	Normal	(*1*,*4*)
6	1987	29, M	NR	China/unknown	Right groin	NR	Subcutaneous mass	Normal	(*1*)
7	1988	22, M	Chinese	Macau/unknown	Spinal cord (T9)	4.0 × 0.2	Lower limb weakness and numbness	4%	(*1*)
8	1988	22, M	Chinese	Macau/unknown	Spinal cord (T8–9)	1.0 × 0.5	Low back pain and urinary incontinence	4%	(*5*)
9	1991	47, F	Chinese	Hong Kong/unknown	Right breast	≈4.5	Right breast mass	2%	(*6*)
10	1996	NR, NR	NR	NR/unknown	Brain (parietal lobe)	NR	NR	NR	(*7*)
11	1998	27, F	Chinese	Guangxi province, China/application of raw frog meat poultices	Right basal ganglia	0.22 × 0.15	Left-sided numbness for 36 mo.	Normal	(*8*)
12	2004	80, F	NR	NR/unknown	Left breast	22 × 5	Left breast	NR	(*9*)

*L, length; NR, not recorded; W, width.

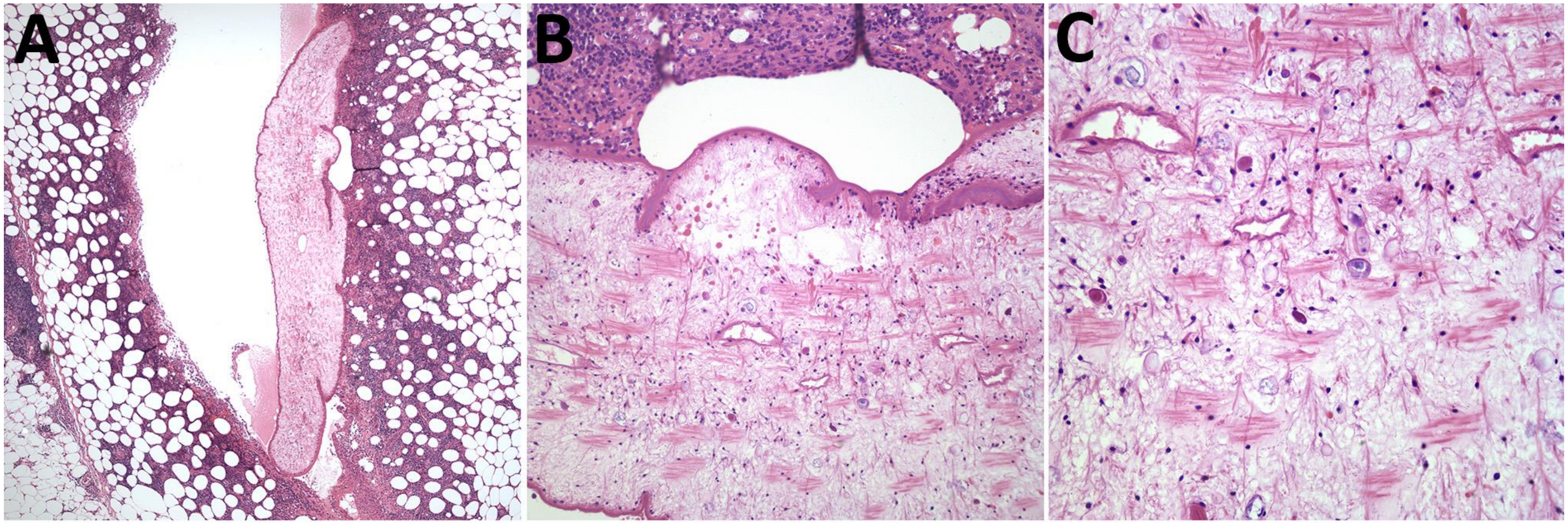

Technical Appendix Figure 1. Hematoxylin-eosin staining of tissue section from lesion in patient 7. A) Scanning view of the lesion (original magnification, 4×). B) Low-power view of the sparganum (original magnification, 20×). C) High-power view of the sparganum (original magnification, 40×).

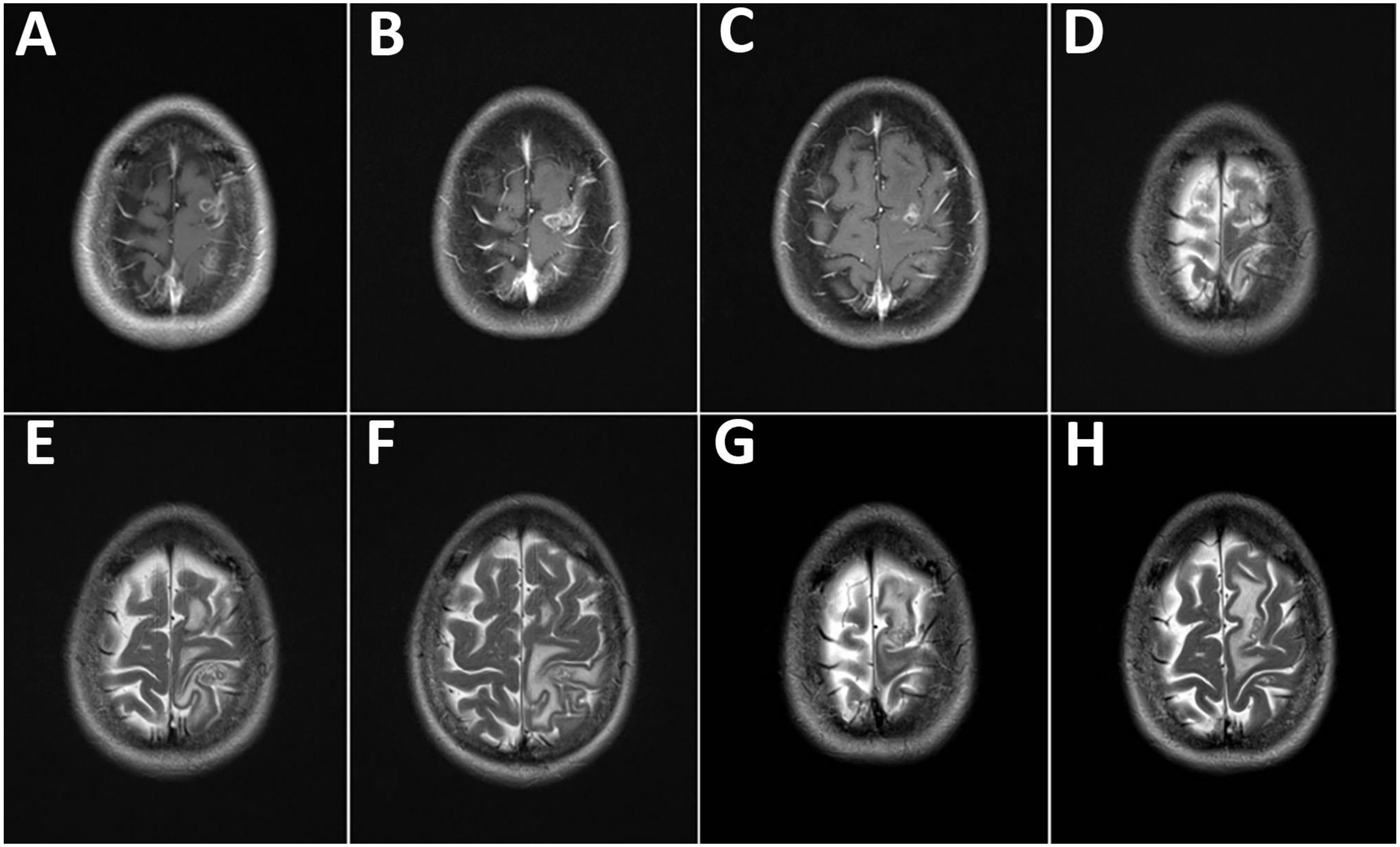

Technical Appendix Figure 2. Serial magnetic resonance imaging (MRIs) brain scans of patient 8. The bottom of the images correspond to the back of the head. A–C) T1-weighted images of initial scan. D–F) T2-weighted images of initial scan showing 3 lesions in the left high parietal and left inferior frontal areas. G–H) Follow-up MRI T2-weighted images 62 days later.
